# Long-term intermittent cold exposure affects peri-ovarian adipose tissue and ovarian microenvironment in rats

**DOI:** 10.1186/s13048-021-00851-8

**Published:** 2021-08-21

**Authors:** Li Zhang, Gaihong An, Shuai Wu, Jing Wang, Danfeng Yang, Yongqiang Zhang, Xi Li

**Affiliations:** Tianjin Institute of Environmental and Operational Medicine, Tianjin, 300050 China

## Introduction

Cold is a significant environmental stress factor. Studies have shown that exposure to cold environments can cause local or whole-body temperatures to decrease, posing a severe threat to overall health [1–3]. Cold exposure has adverse effects on the female reproductive system [4–6], affecting ovarian [7] and uterine [4] functions and hormone secretion [8]. Possible reasons include: imbalance of ET-1 and its receptor expression leads to local tissue microvascular circulatory disturbances [9]; affects follicular development by activating sympathetic nerve activity in the ovary [10, 11]; Cold stress can also cause reproductive hormone disorders, causing uterine arteries to contract, resulting in reduced blood flow [12]. However, the exact mechanisms through which these changes occur have not been well-elucidated.

FSH is a glycoprotein hormone secreted by adenohypophysial gonadotropin cells. Studies have shown that its production is controlled by hypothalamic gonadotropin-releasing hormone (GnRH) and is also regulated by the feedback of estradiol (E2). More than 95% of the E_2_ in circulation is secreted by the ovary, and the growth and development of follicles during each maturation stage require the presence of E_2_. In addition, E_2_ can directly promote the development and maturation of eggs and the growth of ovarian granulocytes, in cooperation with FSH [5]. Follistatin (FST) is a non-steroidal ovarian hormone that regulates the secretion and signal transduction of sex hormones and promotes oocyte maturation and embryonic development. FST contents gradually increase as follicles develop. These steroids, non-steroid hormones, and growth factors act as regulatory factors and constitute the microenvironment that determines follicle growth and development. The ovarian microenvironment plays a vital role in ovarian function and follicle development. However, the effects of cold exposure on ovarian function and the ovarian microenvironment have not been well-elucidated.

Studies have shown that the development and maturation of follicles prior to ovulation are primarily regulated by the central neuroendocrine system and growth factors and hormones found in the local ovarian microenvironment [13, 14]. Peri-ovarian adipose tissue (POAT) is a type of white adipose tissue that surrounds the ovaries of rodents. POAT is known to be involved in the development of gonads and germ cells [13, 14]. Unlike brown adipose tissue (BAT), which produces heat, white adipose tissue (WAT) primarily stores energy and secretes biologically active adipokines. Under certain stimuli (cold exposure [15, 16], exercise, etc), WAT will demonstrate characteristics that are typical of BAT, associated with the increased expression levels of BAT marker genes, such as uncoupling protein 1 (*UCP1*), peroxisome proliferator-activated receptor-γ coactivator-1α (*PGC-1α*), PR-domain-containing 16 (*PRDM16*), and fibronectin type III domain-containing protein (*Fndc5*), in a process known as the “browning of WAT.” The body can achieve non-shivering thermogenesis through the browning of WAT [17]. Research on the browning of WAT under cold exposure conditions has primarily focused on subcutaneous adipose tissue (SAT), such as inguinal WAT (iWAT) [18], whereas changes in visceral adipose tissue (VAT), such as POAT, are rarely reported. Determining whether POAT browns after cold exposure and whether POAT browning affects ovarian function is necessary to better understand the mechanisms that influence the reproductive system in cold environments.

In recent years, many studies have shown that adiponectin (ADPN), leptin (Lep), adenylate-activated protein kinase (AMPK), and other adipokines are involved in ovarian function. High levels of ADPN can regulate the production of ovarian steroid regulatory factors, such as E_2_, FSH, and progesterone (P) [19, 20]. In addition, Lep receptor is expressed in both follicular cells and oocytes of female mice, and Lep activates STAT3 through Lep receptor in the second meiosis, suggesting that Lep may be an important factor in oocyte maturation [21]. In women, the serum level of Lep was correlated with E2 and LH levels, and its level is too high or too low can affect reproductive function [22, 23]. Ovarian microenvironmental regulators, such as FST, also affect adipokines and the browning of WAT. For example, Shan et al. found that FST promotes the browning of WAT, through the *AMPK-PGC-1α-Fndc5* axis [24]. These results hinted the existence of an association between POAT adipokines and ovarian microenvironment regulators. Therefore, whether POAT browns after cold exposure and is regulated by the activation of the *AMPK-PGC-1α-Fndc5* pathway remains uncertain. Additionally, the browning of WAT may be regulated by the levels of E_2_, FSH, and FST in the ovarian microenvironment. Further research on this issue will help us to understand the regulatory mechanisms of ovarian dysfunction that are induced by cold exposure and provide further information regarding the ovarian regulation mechanism. Therefore, the purpose of this study is to explore the effect of cold exposure on reproductive endocrine disruption and its mechanism, and to provide scientific basis for clarifying the potential impact of cold environment on female reproductive system.

## Materials and methods

### Animal experiments and ethical approval

Specific pathogen-free, female, Sprague–Dawley (SD) rats, 8 weeks, weighing 200 ± 10 g, were obtained from Weitong Lihua Experimental Animal Technology Co., Ltd. (Beijing, China) and were housed at 23 ± 1 °C and 45–60% humidity, under a 12-h light-dark cycle, with free access to food and water. The animals were fed in their cages for 2 days, and then vaginal cytological smears were performed, at the same time every morning and evening. A total of 20 rats with regular estrous cycles were randomly divided into two groups: (1) the control group, in which the rats were maintained at room temperature (23 ± 1 °C), for 2 weeks; and (2) the cold exposure group, in which the rats were maintained at − 10 °C, for 4 h each day, for 2 weeks, as previous report [25]. During the cold exposure period of the experimental group, rats in the control group were also fasted and prohibited from consuming water. Within 8 h after the last cold exposure, a surgical procedure was conducted during the diestrus stage. Blood was sampled from the abdominal aorta, after which, the animals were immediately euthanized. The organs of ovaries and POAT were removed from each rat. The blood samples were centrifuged at 3000 rpm for 15 min and stored at − 80 °C. All procedures relating to animal care and use were approved by the Ethics Review Committee of the Institute of Environmental and Operational Medicine (IACUC of AMMS-04-2020-017).

### Body weight and organ coefficients

To determine the effects of cold exposure procedures, the body weights and ovary coefficients were measured before and at the end of the experiment in both groups. The ovaries of each rat were dissected and weighed after euthanasia (wet basis). The following formula was used to calculate the organ coefficients: organ coefficients (%) = $\frac{\mathrm{Organ}\ \mathrm{weight}\ \left(\mathrm{g}\right)}{\mathrm{Body}\ \mathrm{weight}\ \left(\mathrm{g}\right)}\times 100\%$.

### Serum hormonal analysis

The serum levels of FSH, E_2_, P, and testosterone (T) were determined using a radioimmunoassay kit (FuRui Runze Biotechnology, Beijing, China), according to the manufacturer’s instructions.

### Immunohistochemistry

The ovary tissues were Fixed, embeded, sectioned, deparaffinized, rehydrated, and microwaved (850 W) in 0.01 M citrate buffer (pH 6.0) for antigen retrieval 20 min. The sections were then blocked with normal serum and incubated with anti-FSH receptor (FSHR, GB11275–1, Abcam, 1:800) and anti-estrogen receptor β (ERβ) antibodies (GB11268, Abcam, 1:500). Using the Image-Pro Plus 6.0 analysis system (Media Cybernetics, Silver Spring, MD), protein expression was measured, and the regional average optical density value was determined for quantitative analysis.

### Determination of AMH levels in serum

The serum AMH level was determined by enzyme-linked immunosorbent assay (ELISA). The specific experimental steps were performed in strict accordance with the kit instructions [Rat Mueller tube inhibitor/anti-Mueller tube hormone (AMH) ELISA kit (KAMIYA, KT-35857)].

### Ovarian histology and antral follicle counts

The ovaries (*n* = 10) were fixed, processed, stained with eosin and hematoxylin, and examined under a light microscope, to quantify the ovarian follicular reserve. Ten representative sections from each ovary were selected for follicle counting, with each observed section separated by a distance of over 40 μm. Differential follicle counts were determined by referring to previous literature [7, 26, 27]. The follicles were classified as primordial follicles, primary follicles, secondary follicles, antral follicles, or corpus luteum, based on their structural features [3]. Each ovary was cut 10 slices and counted the total number of primordial follicles, primary follicles, secondary follicles, mature follicles and corpus luteum in each sample slice under the microscope.

### Transmission Electron microscopy analysis

POAT samples were obtained and fixed in 10% glutaraldehyde, at 4 °C for 2 h, and then dehydrated through a graded ethanol series and embedded in embedding agent (Wuhan Service Biotechnology CO., LTD, 90529–77-4). Ultrathin sections, 60–80 nm thick, were cut using an ultrathin microtome (Leica, Leica UC7). A Hitachi transmission electron microscope (HT7700) was used to observe and analyze the images.

### RNA extraction and reverse transcription-quantitative polymerase chain reaction (RT-qPCR)

Total RNA was isolated from the ovary and POAT, using RNeasy Mini kit (Qiagen, Valencia, CA, USA), and cDNA was synthesized from 500 ng total RNA, using PrimeScripe RT Master Mix (Perfect Real Time, Takara Bio, Otsu, Japan). Reverse transcription-quantitative real-time-polymerase chain reaction (RT-qPCR) was performed using Step One Plus (Applied Biosystems, Foster City, CA, USA). Template cDNA (5 ng/μl) was mixed with Fast SYBR Green Master Mix (Thermo Fisher Scientific, Waltham, MA, USA), distilled water, and primers (final concentration 500 nM). The reaction was performed at 95 °C for 20 s, followed by 40 cycles at 95 °C for 3 s and 60 °C for 30 s. The primers were designed as Table 1. The relative expression level of each gene was determined using the ΔΔ-C_t_ method, normalized against β-actin expression.

**Table 1:** qRT-RCR primer sequences

Name	Primer sequences
**FST**	5′-CAGCGACAATGCCACGTA-3’
	5′-TGCACACTGCTGGACAGTTTA-3’
**Lep**	5′-CGAGACCTCCTCCATCTGCT-3’
	5′-CTGCTCAAAGCCTCCACCTC-3’
**ADPN**	5′-CTGGGCATCTCTGCCATCA-3’
	5′-CTTGACAAAGCCCTCAGCGATA-3’
**AMPK**	5′-TGAGCTTACAGCTTTACCTGGTTG-3’
	5′-CACTTGACCGAGGTCTGTGGA-3’
**UCP-1**	5′-GTACCCAGCTGTGCAATGAC-3’
	5′-GATGACGTTCCAGGATCCGA-3’
**PGC-1α**	5′-GCACTGACAGATGGAGACGTGA-3’
	5′-TCATTGTAGCTGAGCTGAGTGTTGG-3’
**PRDM16**	5′-ACAAAGGGAAGCCAGCAGAG-3’
	5′-GAGGCGGGAAGAAGGAATG-3’
**Dpt**	5′-AGGGCTCTGACAGACAGTGGAACTA-3’
	5′-ACTGACTCGAAGTAACGGCTTTGG-3’
**HOXC9**	5′-CGGCAGCAAGCACAAAGAG-3’
	5′-ACCGACGGTCCCTAGTTAAATACA-3’
**Fndc5**	5′-ATCATCGTCGTGGTCCTCTTC-3’
	5′-TGGTCTCTGATGCACTCTTGG-3’
**MST**	5′-CGCTACCACGGAAACAATCATT-3’
	5′-GCTTTCCATCCGCTTGCA T-3’
**cyb11a1**	5′-AGAAGCTGGGCAACATGGAGT-3’
	5′-TCACATCCCAGGCAGCTGCATGGT-3’
**cyp19a1**	5′-TAAAAGATGGCACACAAAGAGTGC-3’
	5′-ACCGAGGTTACCTGGATCTGC-3’
**β-actin**	5′- CCTAAGGCCAACCGTGAAAA −3’
	5′- CAGAGGCATACAGGGACAACAC − 3’

### Statistical analysis

All values are presented as the mean ± standard error. SPSS 21.0 statistical software was used for statistical analysis. Kolmogorov Smirnov test (with dallal Wilkinson lilliefer *p* value), D’Agostino and Pearson omnibus normality test and Shapiro Wilk normality test were used to detect whether the data were normal distribution. An independent Student’s *t-*test was performed to determine significant differences between experimental groups. A repeated-measures analysis of variance (ANOVA), followed by a Tukey’s post hoc test, was implemented to compare body weight gains. *P* < 0.05 indicated a significant difference.

## Results

### Effect of cold exposure on body weight and organ coefficient in female rats

**Fig. 1:**
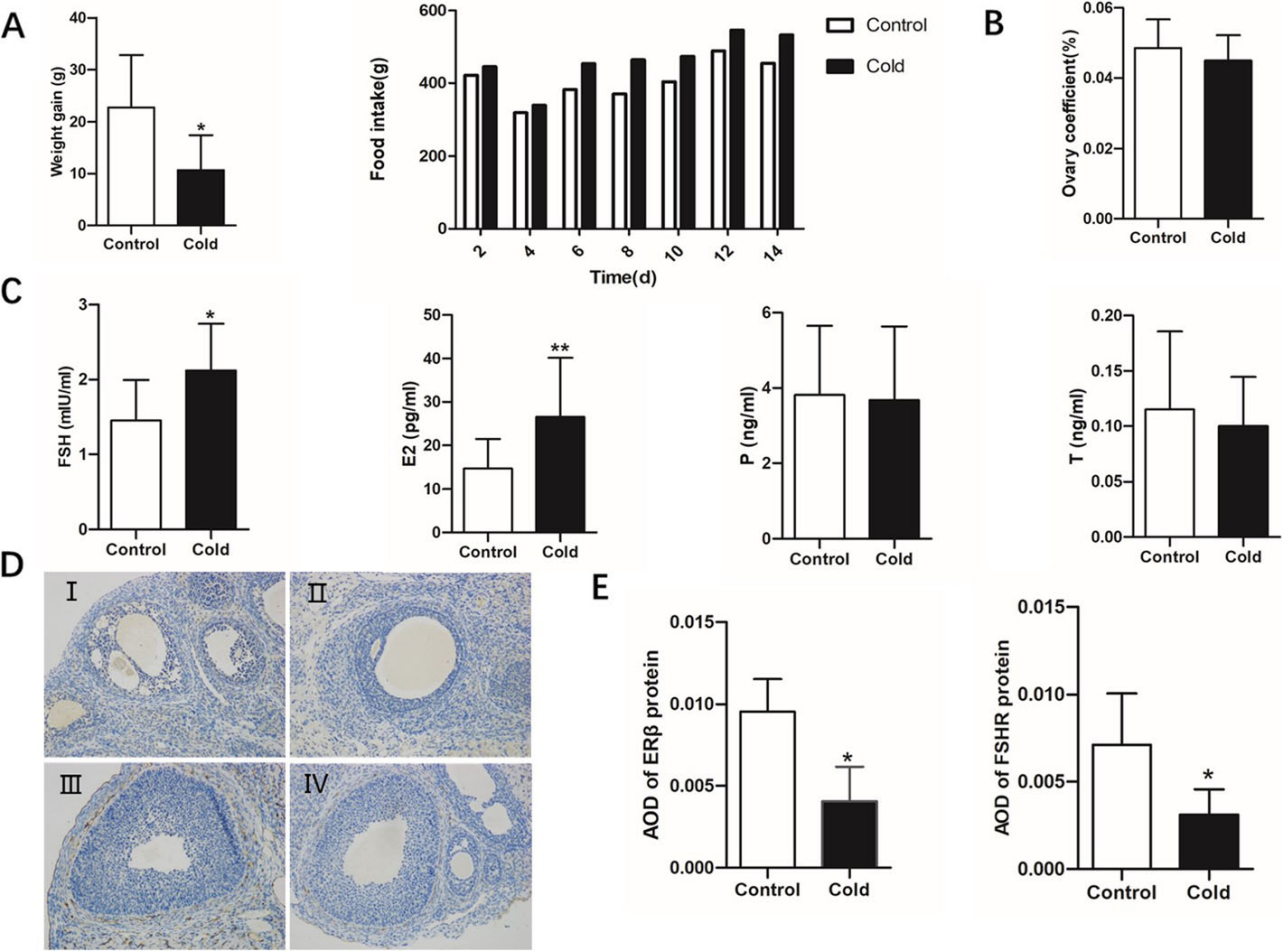
Cold exposure impairs the ovarian function of female rats. **A** Effects of cold exposure on body weight gain and food intake (*n* = 10). **B** Effects of cold exposure on the ovary coefficients (*n =* 10). **C** Serum levels of E2, FSH, P, and T in control and cold-exposed groups of female rats (*n =* 10). **D** and **E** Expression levels of ERβ and FSHR in ovarian tissue in cold-exposed and control groups of female rats (*n* = 6). Values are presented as the mean ± standard error. ^∗^*P* < 0.05 and ^∗∗^*P* < 0.01

Consistent with our previous results, the cold exposure group showed a significantly reduced rate of body weight gain compared with that in the control group, but the food intake of rats in the cold exposure group was higher than that in the control group (Fig. 1A). The ovary coefficients showed no significant difference between the two groups (Fig. 1B). The levels of serum E_2_ and FSH in female rats were significantly higher in the cold exposure group than in the control group, whereas the levels of serum P and T showed no significant differences between the two groups (Fig. 1C). Compared with those in the control group, the protein expression levels of ERβ (Fig. 1D-I and II and E) and FSHR (Fig. 1D-III and IV and E) were significantly down- regulated in the cold exposure group (*P* < 0.05).

### Cold exposure affects follicular development

**Fig. 2:**
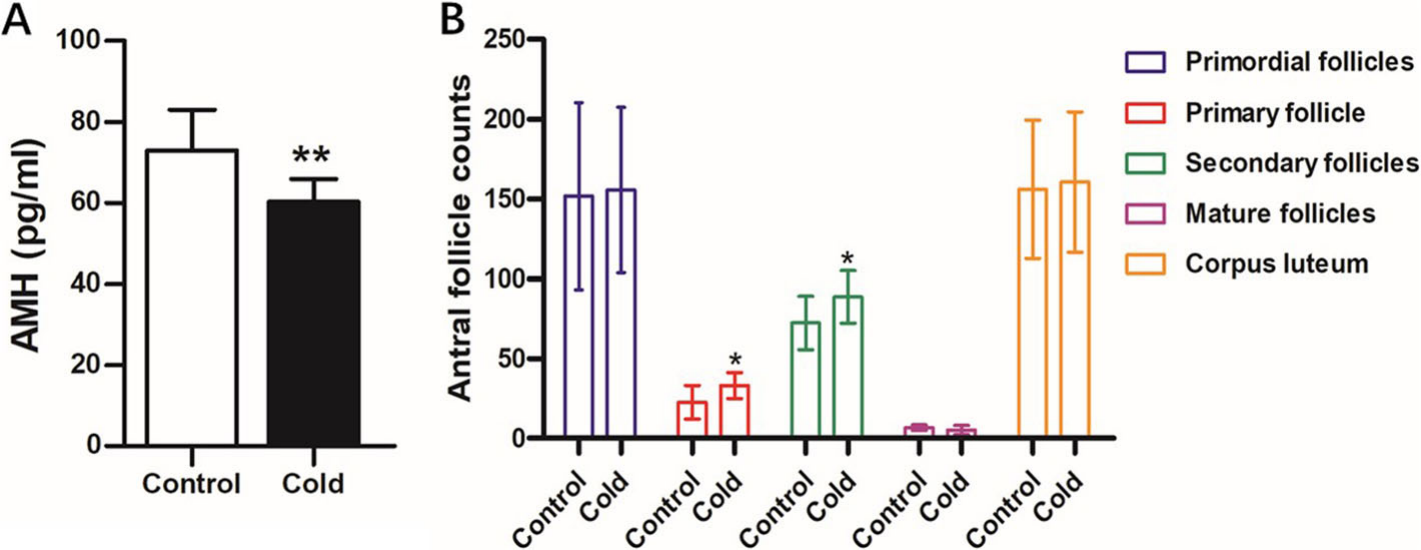
Effect of Cold Exposure on follicular development in female rats. **A** Serum AMH levels in control and cold-exposed groups of female rats (*n =* 10). **B** Effects of cold exposure on ovarian histology and antral follicle counts (*n =* 10)

The results showed that the serum AMH level decreased significantly after cold exposure for 14 days (Fig. 2A). We also counted the follicles on each slice. Statistical analysis showed that the numbers of primary and secondary follicles in the cold-exposed group were significantly higher than those in the control group (*P* < 0.05), whereas the numbers of antral follicles, corpus lutea, and total follicles showed no significant differences (Fig. 2B).

### Cold exposure promotes POAT browning

**Fig. 3:**
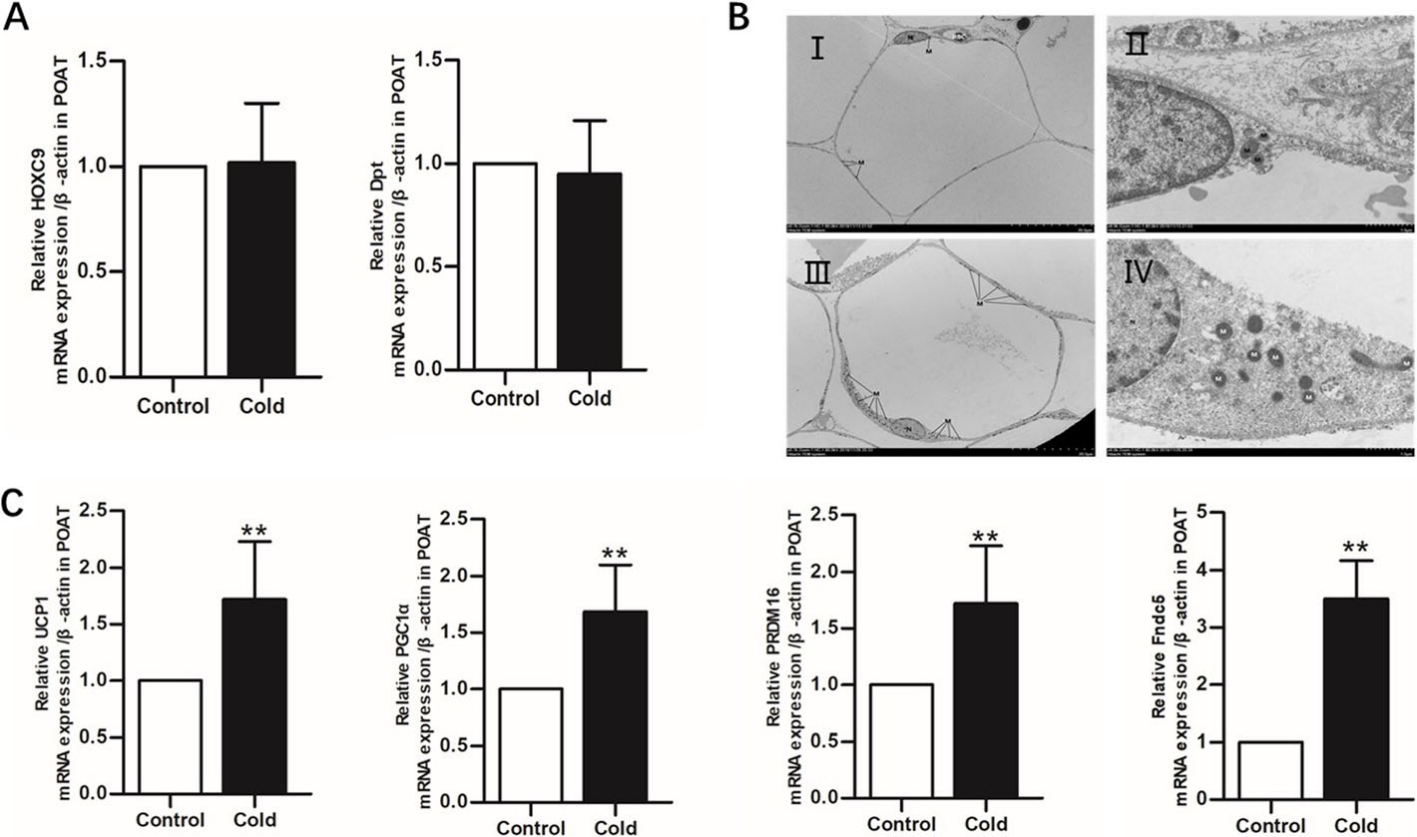
Cold exposure promotes POAT browning. **A** Gene expression levels of *Dpt* and *HOXC9* in cold-exposed and control groups of female rats (*n =* 6). **B** Ultrastructural changes in POAT, assessed by TEM. Control group: (I and II). Cold exposure group: (III and IV). **C** Gene expression levels of *UCP1*, *PGC1α*, *Fndc5*, and *PRDM16* in cold-exposed and control groups of female rats (*n =* 6). Values are presented as the mean ± standard error. ^∗^*P* < 0.05 and ^∗∗^*P* < 0.01

After chronic cold exposure, no significant differences in the expression of WAT-related genes, such as *Dpt* and *HOXC9,* were observed between the cold exposure group and the control group (Fig. 3A). However, the expression levels of four BAT-related genes, including *UCP1, PGC-1α, Fndc5*, and *PRDM16,* increased significantly in the cold-exposed group compared with those in the control group (*P* < 0.05), as assessed by RT-qPCR on RNA isolated from POAT (Fig. 3C). When comparing the organelles and cell morphologies between the control and cold exposure groups, no obvious lipid droplets were observed in samples from either group, whereas the number of mitochondria in the cold-exposed group was significantly higher than that in the control group. These results showed that cold exposure had a significant effect on the number of mitochondria. These findings confirmed that cold exposure could induce POAT browning (Fig. 3B).

### POAT browning and the local ovarian microenvironment

**Fig. 4:**
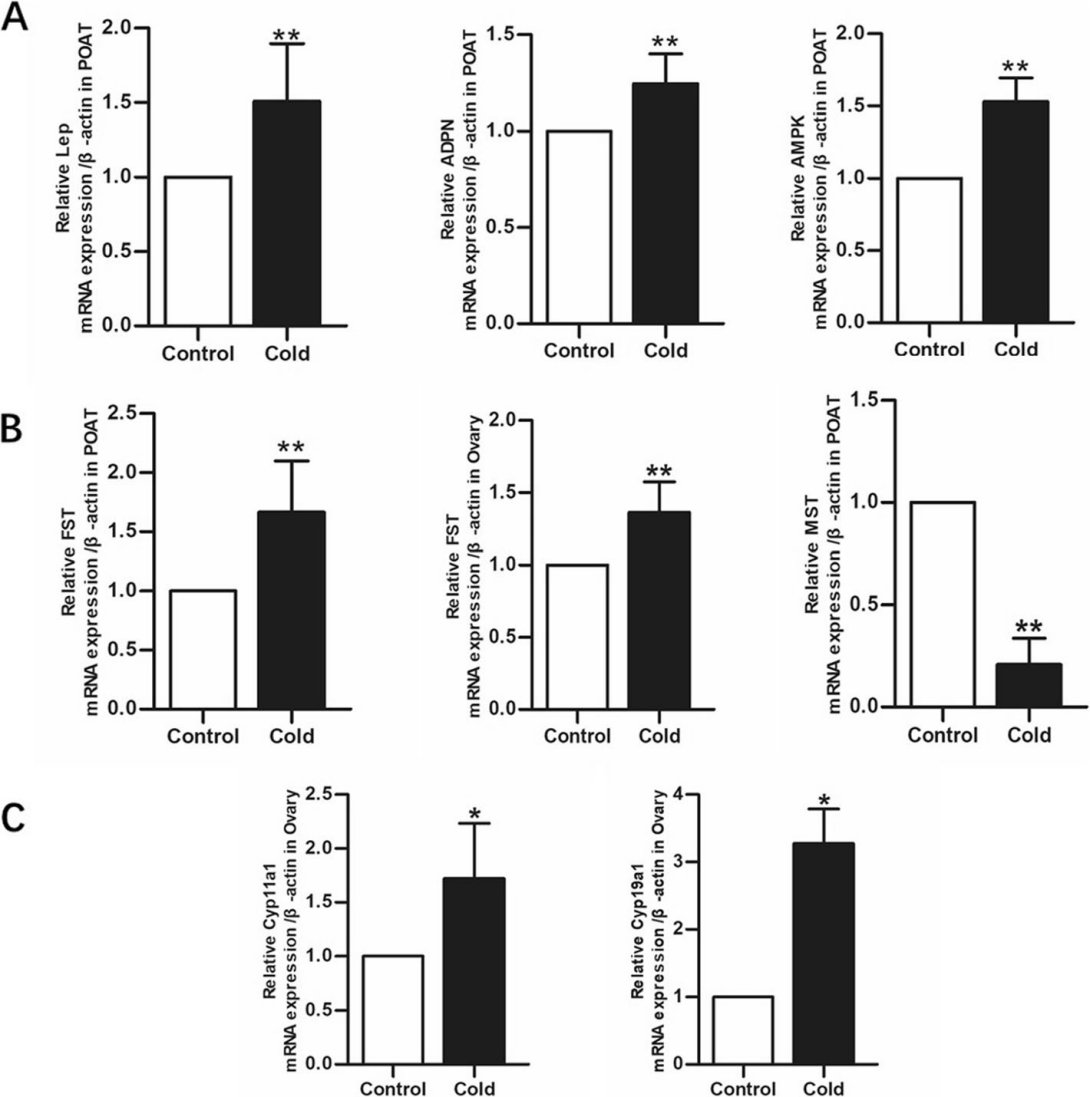
Effects of cold exposure on the local ovarian microenvironment of female rats. **A** Gene expression levels of *Lep*, *ADPN,* and *AMPK* in both groups (*n =* 6). **B** Gene expression levels of *FST* in the ovary and POAT of both groups (*n =* 6). **C** Gene expression levels of *cyb11b2* and *cyp19a1* in the ovary of both groups (*n =* 6). Values are presented as the mean ± standard error. ^∗^*P* < 0.05 and ^∗∗^*P* < 0.01

Our results showed that the gene expression levels of *AMPK, Lep,* and *ADPN* in the cold exposure group were significantly upregulated compared with those in the control group (Fig. 4A). The *FST* mRNA levels in POAT and ovarian tissue were significantly increased in the cold exposure group compared with those in the control group, whereas the *MST* mRNA level in POAT showed a significant decrease in the cold exposure group compared with that in the control group (Fig. 4B). The *cyb11a1* and *cyp19a1* mRNA levels in the ovary showed substantial increases in the cold exposure group compared with those in the control group (Fig. 4B).

## Discussion

Our results showed that body weight gains decreased and changes were observed in the levels of serum sex hormones and ovarian hormone receptors, although the ovarian organ index did not change significantly. Additionally, serum AMH levels decreased and the numbers of primary and secondary follicles increased, after 2 weeks of cold exposure. AMH is one of the most effective and sensitive serological indicators of ovarian function [28], which directly reflects the ovarian reserve function. AMH can inhibit the recruitment of primordial follicles and the early growth of follicles by activating AMH receptor II, preventing premature follicle failure, and AMH concentrations change with age instead of undergoing menstruation-related periodic changes. Follicle counting (FC) is used to examine the different types of follicles during folliculogenesis. Currently, AMH and FC are used to evaluate the development of follicles at different stages under various stimulation conditions [7, 26, 27]. The results of this study and our previous studies [29] have suggested that cold exposure can affect the metabolism of rats, resulting in reductions in weight gain and damage to the ovarian structure and reserve function, which affects follicular development.

POAT is a type of WAT that clings to the ovary. In mammals, adipose tissue exists as BAT, WAT, and beige adipose tissue. BAT produces heat, whereas WAT stores energy and participates in endocrine functions. Studies have shown that to promote bodily adaptations to a cold environment under cold exposure conditions, subcutaneous WAT, such as inguinal fat (iWAT), will brown [30]. However, the existence of gonadal adipose tissue Browning remains controversial [31]. In this study, our results suggested that after cold exposure, the number of adipocyte mitochondria in POAT increased significantly, and specific genes associated with the browning of WAT, such as *UCP1*, *PGC*-*1α*, *PRDM16*, and *Fndc5*, were significantly upregulated. Two weeks of continuous cold exposure was found to induce browning in POAT.

The changes that occur in the POAT after browning and the effects of these changes on the peri-ovarian microenvironment, the ovarian microenvironment, and ovarian function have not been explored. We found that after POAT browning, the expression levels of APDN, Lep, and AMPK were significantly increased, similar to the effects observed during iWAT browning [9, 32]. Our results suggested that the peri-ovarian microenvironment changes along with POAT browning.

Ovarian estrogen, which is regulated by endocrine factors from the central nervous system, can affect the differentiation of female ovarian cells and plays an important role in the regulation of follicular development. E2 is the most biologically active form of estrogen. P and T are intermediate products of E_2_ synthesis. Studies have shown that E_2_ deficiency (such as in an E_2_ synthesis rate-limiting enzyme knockout) causes mouse follicle development to stop during the sinusoidal follicle stage, and these symptoms can be relieved by the administration of exogenous E_2_ [5]. Simultaneously, other studies have shown that E_2_ exerts anti-apoptotic functions and plays roles in cell protection and the regulation of lipid metabolism [33]. FST is a nonsteroidal hormone expressed in the ovary, brain, pituitary, and adrenal gland and acts as an important local regulatory factor for ovarian follicles [34]. FST regulates FSH secretion [4], promotes follicle maturation [35], and promotes embryo development. Jorgez et al. [35] found that mature follicles and oocytes in *FST*-knockout adult animals presented delayed maturation and development, and ovarian activity was terminated in advance. The administration of exogenous FST to bovine embryos during the cleavage stage could significantly improve the early cleavage rate. Regulatory factors, such as E_2_, FSH, and FST, together constitute the ovarian microenvironment. Increasingly, researchers believe that in addition to the central nervous system regulation which is the most important regulation way, ovarian function is also regulated by both the ovarian and peri-ovarian microenvironment. Therefore, we speculate that the ovarian and peri-ovarian microenvironments may also play important roles in the follicular dysplasia induced by cold exposure.

Previous studies have shown that APDN, Lep, AMPK, and other adipokines can exert effects through autocrine, paracrine, and endocrine mechanisms. On the one hand, adipokines affect the development of follicles, and on the other hand, they affect lipid accumulation and adipose tissue metabolism in the ovarian microenvironment. APDN plays an important role in follicular development by regulating cytochrome P450 cholesterol side-chain lyase (Cyplla1) and promoting E2 synthesis [36]. Leptin, as an important adipokine, can act on ovarian tissue by regulating the expression of CYP17 and CYP19 and affecting the sensitivity of granulosa cells to FSH [37]. In POAT-excised mice, APDN contents decreased, and the expression levels of *Cyplla1, Cyp19,* and other genes were suppressed, resulting in decreased E_2_ expression in the ovarian microenvironment. Leptin can act on the ovary to promote the sensitivity of granulosa cells to FSH [37]. Wang et al. [38] have demonstrated that POAT excision can cause a decrease in the Lep level of the ovarian microenvironment, which ultimately causes the granulosa cells to become less sensitive to FSH. In addition, in *Lep*-knockout mice, estrogen synthesis is reduced [39]. These results showed that changes in APDN and Lep levels could affect the expression levels of E_2_ and FSH. Therefore, we detected the serum levels E_2_, FSH, T, and P, and examined the gene expression levels of ERβ, FSHR, and the rate-limiting enzymes of the E_2_ synthesis pathway in rat ovaries after cold exposure. The results showed no differences in serum P and T levels, whereas serum E_2_ and FSH levels increased, ovarian ERβ and FSHR expression decreased, and rate-limiting enzymes in the E_2_ synthesis pathway, such as *Cyplla1* and *Cyp19a1,* were upregulated. Ahima et al. [1] also found that Lep treatment could significantly increase the numbers of primordial follicles, primary follicles, secondary follicles, and mature follicles in obese female mice, suggesting that Lep administration can improve the physiological functions of the ovary, to a certain extent. In this study, Lep upregulation appeared to play a similar role. Although the total numbers of follicles in rats did not change significantly after cold exposure, the numbers of primary and stimulated follicles increased significantly. These results suggested that APDN and Lep may affect follicular development and the ovarian microenvironment after POAT browning.

The development and maturation of follicles require energy, provided by various substrates (glucose, proteins, and lipids). AMPK is involved in energy metabolism, and decreased AMPK expression leads to a decrease in lipid accumulation in the ovarian microenvironment and the compensatory activation of the fatty acid biosynthesis pathway in the ovary [38]. Simultaneously, AMPK participates in the browning of adipose tissue, through the AMPK-PGC1α-Fndc5 pathway. Shan et al. found that the browning of WAT occurred in myostatin (*MST*)-knockout rats. The deletion of *MST* led to the increased expression of AMPK protein, and AMPK indirectly activated the expression of *PGC-1α* and *Fndc5*. *PGC-1α* and *Fndc5* are related genes that promote the expression of BAT and beige adipose tissue [8, 40]. MST is a negative regulator of skeletal muscle and inhibits AMPK protein expression [41, 42], and MST is inhibited by FST [43]. FST is closely related to the browning of adipose tissue [24, 44]. Singh et al. [44] found that when FST is overexpressed in transgenic mice, the quality of BAT increased and the expression levels of proteins associated with BAT and beige fat increased in WAT. Exogenous FST can promote the expression of *Fndc5* in mouse cells [24]. In this study, after the browning of POAT, the expression level of *MST* was downregulated in POAT, and FST levels in the POAT and ovary were upregulated. These results suggested that FST may promote the browning of WAT by inhibiting MST and activating the AMPK-PGC1α-Fndc5 pathway.

Therefore, we believe that cold exposure causes abnormal follicular development, damages ovarian function, and induces POAT browning. POAT browning relieves the adverse effects associated with cold exposure on ovarian function, to a certain extent. Although we were unable to determine the exact timing of POAT browning and ovarian microenvironment changes, we tend to believe that after cold stimulation, mutual adjustments occur in the levels of adipokines induced by POAT browning and ovarian regulatory factors. This process constitutes one of the body’s compensation adjustment mechanisms following cold exposure. In addition, WAT, such as subcutaneous adipose tissue (iWAT) and adipose organ tissue (peri-dimensional adipose tissue), can directly sense temperature and generate heat, inducing the increased expression of *UCP1* and *PRDM16* (by 2–3-fold) [45]. We speculate that the effects of POAT browning on the local ovarian microenvironment, due to changes in adipokines, may represent a regulatory mechanism, independent of central regulation. However, this hypothesis requires further verification.

Our study has several limitations. First, the relationship between POAT adipokines and the local ovarian microenvironment has not been fully elucidated and requires further investigation. Second, we haven’t explored the function of follicles, especially mature follicles. Therefore, the compensatory effect of POAT browning needs further study. Third, due to the lack of cold exposure intensity gradient verification, the potential and limits of POAT browning to provide compensatory protection for ovarian function under cold exposure conditions have not yet been elucidated. Fourthly, since it is impossible to separate stress response from cold exposure in the design of this study, this model actually verifies the effect of cold exposure and related stress factors caused by cold exposure on ovarian physiological function. Future research remains necessary to clarify these issues. We hope that our research can stimulate interest in this field.

## Conclusions

In summary, this research showed that cold exposure can cause abnormal follicle development and promote the browning of POAT. POAT browning is accompanied by the activation of the AMPK-PGC1α-Fndc5 pathway. The effects of POAT browning on the local microenvironment of the ovary is hypothesized to represent a mechanism through which the body compensates for the regulation of ovarian function under cold exposure conditions.

## Supplementary Information

**Additional file 1.**

## Data Availability

The datasets used and/or analyzed during the current study are available from the publicly available SEER database.

## Abbreviations

3β-HSD: 3β-hydroxysteroid synthase
ADPN: Adiponectin
AMH: Anti-Mueller tube hormone
AMPK: Adenylate-activated protein kinase
BAT: Brown adipose tissue
Cyp19: Aromatase
Cyplla1: Cytochrome P450 cholesterol side chain lyase
Dpt: Dermatopontin
E2: Estradiol
FC: Follicle counting
Fndc5: Fibronectin Type III Domain containing Protein5
FSH: Follicle-stimulating hormone
FST: Follistatin
Hoxc9: Homeobox C9
iWAT: Inguinal WAT
LEP: Leptin
MST: Myostatin
P: Progesterone
PGC-1α: Peroxisome proliferators activated receptor-γ coactivator-1α
POAT: Peri-ovarian adipose tissue
PRDM16: PR domain containing 16
STAR: Acute regulatory protein
TEM: Transmission electron microscopy
UCP1: Uncoupling protein 1
WAT: White adipose tissue

## References

[1] Ahima RS, Dushay J, Flier SN, Prabakaran D, Flier JS (1997). Leptin accelerates the onset of puberty in normal female mice. J Clin Invest.

[2] Ahmadi E, Nazari H, Hossini-Fahraji H (2019). Low developmental competence and high tolerance to thermal stress of ovine oocytes in the warm compared with the cold season. Trop Anim Health Prod.

[3] Banerjee S, Banerjee S, Saraswat G, Bandyopadhyay SA, Kabir SN (2014). Female reproductive aging is master-planned at the level of ovary. PLoS One.

[4] Besecke LM, Guendner MJ, Sluss PA, Polak AG, Woodruff TK, Jameson JL (1997). Pituitary follistatin regulates activin-mediated production of follicle-stimulating hormone during the rat estrous cycle. Endocrinology..

[5] Britt KL, Findlay JK (2003). Regulation of the phenotype of ovarian somatic cells by estrogen. Mol Cell Endocrinol.

[6] Cankar K, Music M, Finderle Z (2016). Cutaneous microvascular response during local cold exposure - the effect of female sex hormones and cold perception. Microvasc Res.

[7] Chen LJ, Yang ZX, Wang Y, Du L, Li YR, Zhang NN (2019). Single xenotransplant of rat brown adipose tissue prolonged the ovarian lifespan of aging mice by improving follicle survival. Aging Cell.

[8] Chen YY, Yan Y, Zhao Z, Shi MJ, Zhang YB (2016). Bofutsushosan ameliorates obesity in mice through modulating PGC-1α expression in brown adipose tissues and inhibiting inflammation in white adipose tissues. Chin J Nat Med.

[9] Wang D, Cheng X, Fang H, Ren Y, Li X, Ren W (2020). Effect of cold stress on ovarian & uterine microcirculation in rats and the role of endothelin system. Reprod Biol Endocrinol.

[10] Fiedler J, Jara P, Luza S, Dorfman M, Grouselle D, Rage F (2006). Cold stress induces metabolic activation of thyrotrophin-releasing hormone-synthesising neurones in the magnocellular division of the hypothalamic paraventricular nucleus and concomitantly changes ovarian sympathetic activity parameters. J Neuroendocrinol.

[11] Jing X, Peng Q, Hu R, Wang H, Yu X, Degen A (2017). Effect of supplements during the cold season on the reproductive system in prepubertal Tibetan sheep ewes. Anim Sci J.

[12] Meidan R, Levy N (2007). The ovarian endothelin network: an evolving story. Trends Endocrinol Metab.

[13] Mishra SR, Thakur N, Somal A, Parmar MS, Reshma R, Rajesh G (2016). Expression and localization of fibroblast growth factor (FGF) family in buffalo ovarian follicle during different stages of development and modulatory role of FGF2 on steroidogenesis and survival of cultured buffalo granulosa cells. Res Vet Sci.

[14] Silva JR, van den Hurk R, de Matos MH, dos Santos RR, Pessoa C, de Moraes MO (2004). Influences of FSH and EGF on primordial follicles during in vitro culture of caprine ovarian cortical tissue. Theriogenology..

[15] Vargovic P, Manz G, Kvetnansky R (2016). Continuous cold exposure induces an anti-inflammatory response in mesenteric adipose tissue associated with catecholamine production and thermogenin expression in rats. Endocr Regul.

[16] Xu Z, You W, Zhou Y, Chen W, Wang Y, Shan T (2019). Cold-induced lipid dynamics and transcriptional programs in white adipose tissue. BMC Biol.

[17] Kajimura S, Seale P, Spiegelman BM (2010). Transcriptional control of brown fat development. Cell Metab.

[18] Seale P, Conroe HM, Estall J, Kajimura S, Frontini A, Ishibashi J (2011). Prdm16 determines the thermogenic program of subcutaneous white adipose tissue in mice. J Clin Invest.

[19] Cheng L, Shi H, Jin Y, Li X, Pan J, Lai Y (2016). Adiponectin deficiency leads to female subfertility and ovarian dysfunctions in mice. Endocrinology..

[20] Landry D, Paré A, Jean S, Martin LJ (2015). Adiponectin influences progesterone production from MA-10 Leydig cells in a dose-dependent manner. Endocrine..

[21] Matsuoka T, Tahara M, Yokoi T, Masumoto N, Takeda T, Yamaguchi M (1999). Tyrosine phosphorylation of STAT3 by leptin through leptin receptor in mouse metaphase 2 stage oocyte. Biochem Biophys Res Commun.

[22] Lin KC, Sagawa N, Yura S, Itoh H, Fujii S (2005). Simultaneous increases of leptin and gonadotropin-releasing hormone following exogenous estrogen administration in women with normally menstrual cycle. Endocr J.

[23] Shafi R, Afzal MN (2008). Status of serum leptin levels in females with infertility. Saudi Med J.

[24] Shan T, Liang X, Bi P, Kuang S (2013). Myostatin knockout drives browning of white adipose tissue through activating the AMPK-PGC1α-Fndc5 pathway in muscle. FASEB J.

[25] Xu T, Li X, Yang L, Zhang Y, Zhang L, Guo Z (2018). Impact of cold exposure on the reproductive function in female rats. Biomed Res Int.

[26] Huang J, Ding Y, Li Z (2019). The regulation of the follicular synchronization and sensitivity of rats with PCOS by AMH during prolonged pituitary downregulation. Gene..

[27] Parlakgumus HA, Aka Bolat F, Bulgan Kilicdag E, Simsek E, Parlakgumus A (2014). Atorvastatin for ovarian torsion: effects on follicle counts, AMH, and VEGF expression. Eur J Obstet Gynecol Reprod Biol.

[28] Depmann M, van Disseldorp J, Broer SL, Eijkemans MJ, Laven JS, Visser JA (2016). Fluctuations in anti-Müllerian hormone levels throughout the menstrual cycle parallel fluctuations in the antral follicle count: a cohort study. Acta Obstet Gynecol Scand.

[29] Riquelme R, Ruz F, Mayerhofer A, Lara HE (2019). Role of ovarian sympathetic nerves and cholinergic local system during cold stress. J Endocrinol.

[30] Squicciarini V, Riquelme R, Wilsterman K, Bentley GE, Lara HE (2018). Role of RFRP-3 in the development of cold stress-induced polycystic ovary phenotype in rats. J Endocrinol.

[31] Dempersmier J, Sambeat A, Gulyaeva O, Paul SM, Hudak CS, Raposo HF (2015). Cold-inducible Zfp516 activates UCP1 transcription to promote browning of white fat and development of brown fat. Mol Cell.

[32] Lin Y, Li X, Zhang L, Zhang Y, Zhu H, Zhang Y (2016). Inhaled SiO (2) nanoparticles blunt cold-exposure-induced WAT-browning and metabolism activation in white and brown adipose tissue. Toxicol Res (Camb).

[33] Lucas TF, Pimenta MT, Pisolato R, Lazari MF, Porto CS (2011). 17β-estradiol signaling and regulation of Sertoli cell function. Spermatogenesis..

[34] Köninger A, Schmidt B, Damaske D, Birdir C, Enekwe A, Kimmig R (2017). Follistatin during pregnancy and its potential role as an ovarian suppressing agent. Eur J Obstet Gynecol Reprod Biol.

[35] Jorgez CJ, Klysik M, Jamin SP, Behringer RR, Matzuk MM (2004). Granulosa cell-specific inactivation of follistatin causes female fertility defects. Mol Endocrinol.

[36] Richards JS, Liu Z, Kawai T, Tabata K, Watanabe H, Suresh D (2012). Adiponectin and its receptors modulate granulosa cell and cumulus cell functions, fertility, and early embryo development in the mouse and human. Fertil Steril.

[37] Kitawaki J, Kusuki I, Koshiba H, Tsukamoto K, Honjo H (1999). Leptin directly stimulates aromatase activity in human luteinized granulosa cells. Mol Hum Reprod.

[38] Wang HH, Cui Q, Zhang T, Guo L, Dong MZ, Hou Y (2017). Removal of mouse ovary fat pad affects sex hormones, folliculogenesis and fertility. J Endocrinol.

[39] Vaira S, Yang C, McCoy A, Keys K, Xue S, Weinstein EJ (2012). Creation and preliminary characterization of a leptin knockout rat. Endocrinology..

[40] Boström P, Wu J, Jedrychowski MP, Korde A, Ye L, Lo JC (2012). A PGC1-α-dependent myokine that drives brown-fat-like development of white fat and thermogenesis. Nature..

[41] Fife E, Kostka J, Kroc Ł, Guligowska A, Pigłowska M, Sołtysik B (2018). Relationship of muscle function to circulating myostatin, follistatin and GDF11 in older women and men. BMC Geriatr.

[42] Iskenderian A, Liu N, Deng Q, Huang Y, Shen C, Palmieri K (2018). Myostatin and activin blockade by engineered follistatin results in hypertrophy and improves dystrophic pathology in mdx mouse more than myostatin blockade alone. Skelet Muscle.

[43] Lee SJ (2007). Quadrupling muscle mass in mice by targeting TGF-beta signaling pathways. PLoS One.

[44] Singh R, Braga M, Reddy ST, Lee SJ, Parveen M, Grijalva V (2017). Follistatin targets distinct pathways to promote Brown adipocyte characteristics in Brown and White adipose tissues. Endocrinology..

[45] Ye L, Wu J, Cohen P, Kazak L, Khandekar MJ, Jedrychowski MP (2013). Fat cells directly sense temperature to activate thermogenesis. Proc Natl Acad Sci U S A.

